# Supplementary material for: A Case Study for the Recovery of Authentic Microbial Ancient DNA from Soil Samples
Source: Microorganisms. 2022 Aug 10;10(8):1623. doi: 10.3390/microorganisms10081623 (PMC9414430; doi:10.3390/microorganisms10081623)
Supplement: Supplementary file 1 [file microorganisms-10-01623-s001.zip › Supplementary_Figures_Tables_legends.pdf]

Supporting Figures

Figure S1. DNA fragment length distributions of collapsed reads of EBCs1 and EBCs2, extracted with five DNA extraction methods and pre-processed using eight bioinformatic pipelines. Each color indicates a different extraction method and bioinformatic pipeline.

Figure S2. PCoA plots of Bray-Curtis and Jaccard dissimilarity matrix calculated using microbial community species-level of data sets. PCoA plots of Bray-Curtis (A) and Jaccard (B) dissimilarity matrix calculated using microbial community species-level of collapsed reads from the 160 contaminated data sets. PCoA plots of Bray-Curtis (C) and Jaccard (D) dissimilarity matrix calculated using microbial community at species-level of collapsed reads from the 160 pre-filtered and post-filtered decontaminated data sets. PCoA plots of Bray-Curtis (E) and Jaccard (F) dissimilarity matrix calculated using microbial community at species-level of collapsed reads from the 140 post-filtered decontaminated data sets. All data sets were rarefied to 1000 sequence reads for the analysis. Samples are indicated with different colors and pre-filtered samples are circled in red.

Figure S3. Taxonomic profiles at phylum level of each sample extracted with five DNA extraction methods and pre-processed using eight bioinformatic pipelines. Profiles for A) Archaea, B) Bacteria and C) Eukaryota domains were obtained by aligning collapsed reads (left) and non-collapsed reads (right) to SILVA SSU 132 as reference database.

Figure S4. Taxonomy profiles for Bacteria domain at genus level of collapsed reads of the four samples pre-processed with the selected bioinformatic pipeline (AdapterRemoval v2, 55kx, deduplication of exact sequences) using A) SILVA SSU 132 B) archaeal and bacterial genomes at complete, chromosome and scaffold-level from the RefSeq database, C) NCBI nucleotide

BLAST database November/2019 and D) Genome Taxonomy Database/release95, as reference databases.

Figure S5. Comparison of alpha diversity indices (Observed features and Shannon's diversity) of data sets obtained from each sample extracted with five DNA extraction methods, pre-processed with the selected bioinformatic pipeline and taxonomically classified using Refseq, NT and GTDB databases.

Supporting Tables legends

Table S1. Effects of DNA extractions and preprocessing pipelines on general sequence statistics. A) General statistics and B) number of collapsed reads with high-quality score (>Q30), from the 232 data sets obtained from each sample extracted with five DNA extraction methods and pre-processed using eight bioinformatics pipelines. C) Pairwise Tukey HSD *post hoc* test of the effect of preprocessing software (AdapterRemoval v2 and FastP), and low-complexity thresholds (none, 30%, 55% and 70%) on the number of sequences and average length, and the effect of deduplication steps on duplication levels, of the 232 data sets obtained from each sample extracted with five DNA extraction methods and pre-processed using eight bioinformatics pipelines. Data was obtained from FastQC reports (v.0.11.7) and visualize using multiqc (v1.0.dev0).

Table S2. Taxonomic classification of the data sets using SILVA 132 database. Summary of taxonomic classification grouped by domain of the collapsed reads of A) 160 contaminated and decontaminated sample data sets, and B) 72 EBCs data sets, obtained using five DNA extraction methods, pre-processed using eight bioinformatics pipelines, and classified using SILVA SSU 132 as the reference database. List of taxa at species-level identified in collapsed

reads of C) 160 contaminated and D) decontaminated sample data sets, obtained using five DNA extraction methods, pre-processed using eight bioinformatics pipelines, and classified using SILVA SSU 132 as the reference database. E) List of contaminant taxa identified in collapsed reads and non-collapsed reads of 72 EBCs data sets. F) List of taxa at species-level identified in non-collapsed reads of the 160 decontaminated sample data sets obtained using five DNA extraction methods, pre-processed using eight bioinformatics pipelines, and classified using SILVA SSU 132 as the reference database.

Table S3. Effects of DNA extractions and preprocessing pipelines on sample diversity indices. A) Alpha diversity indices (observed features and Shannon's index) of 160 decontaminated sample data sets obtained using five DNA extraction methods, pre-processed using eight bioinformatics pipelines, classified using SILVA SSU 132 as the reference database, and rarefied at 1000 sequences depth. B) Alpha diversity (observed features and Shannon's indices) significant differences (Kruskal-Wallis H test, p-value < 0.05) between contaminated and decontaminated data sets, extraction protocols, preprocessing software, low-complexity thresholds, deduplication values, and samples. C) Beta diversity significant differences (PERMANOVA) between contaminated and decontaminated data sets, collapsed and non-collapsed reads, extraction methods, preprocessing software, low-complexity threshold, and deduplication values. D) The ANCOM results for species that differed significantly in abundance levels between decontaminated data sets obtained from collapsed reads and non-collapsed reads.

Table S4. Taxonomic classification of data sets using four different databases. A) Number of assigned collapsed reads to bacterial genus using four reference databases. Bacterial taxonomic classification at genus level of collapsed reads from 20 decontaminated data sets pre-processed

with the selected pipeline (post-filtered 55 kx) using B) SILVA SSU 132, C) Refseq, D) NT and E) GTDB as reference databases. F) Alpha diversity indices of 20 decontaminated data sets pre-processed with the selected pipeline (post-filtered 55 kx) classified using SILVA SSU 132, Refseq, NT and GTDB as reference databases, and their significant differences (Kruskal-Wallis H test, p-value < 0.05) between databases. G) Beta diversity significant differences between each database (PERMANOVA, p-value < 0.050).

Table S5. DNA damage analysis of the 29 data sets pre-processed with the selected pipeline (post-filtered 55 kx) calculated with HOPS software using three reference databases. Summary of total number of ancient and default reads for data sets taxonomically classified using A) GTDB, B) NT and C) Refseq, as reference database. Number of damaged and default reads per species using D) GTDB, E) NT and F) Refseq, as reference database. G) DNA damage patterns of the taxa Acidobacteria in Sample 1 extracted with five extraction methods, calculated against the GTDB as reference database.
