# Supplementary figures and images for: A Case Study for the Recovery of Authentic Microbial Ancient DNA from Soil Samples
Source: Microorganisms. 2022 Aug 10;10(8):1623. doi: 10.3390/microorganisms10081623 (PMC9414430; doi:10.3390/microorganisms10081623)

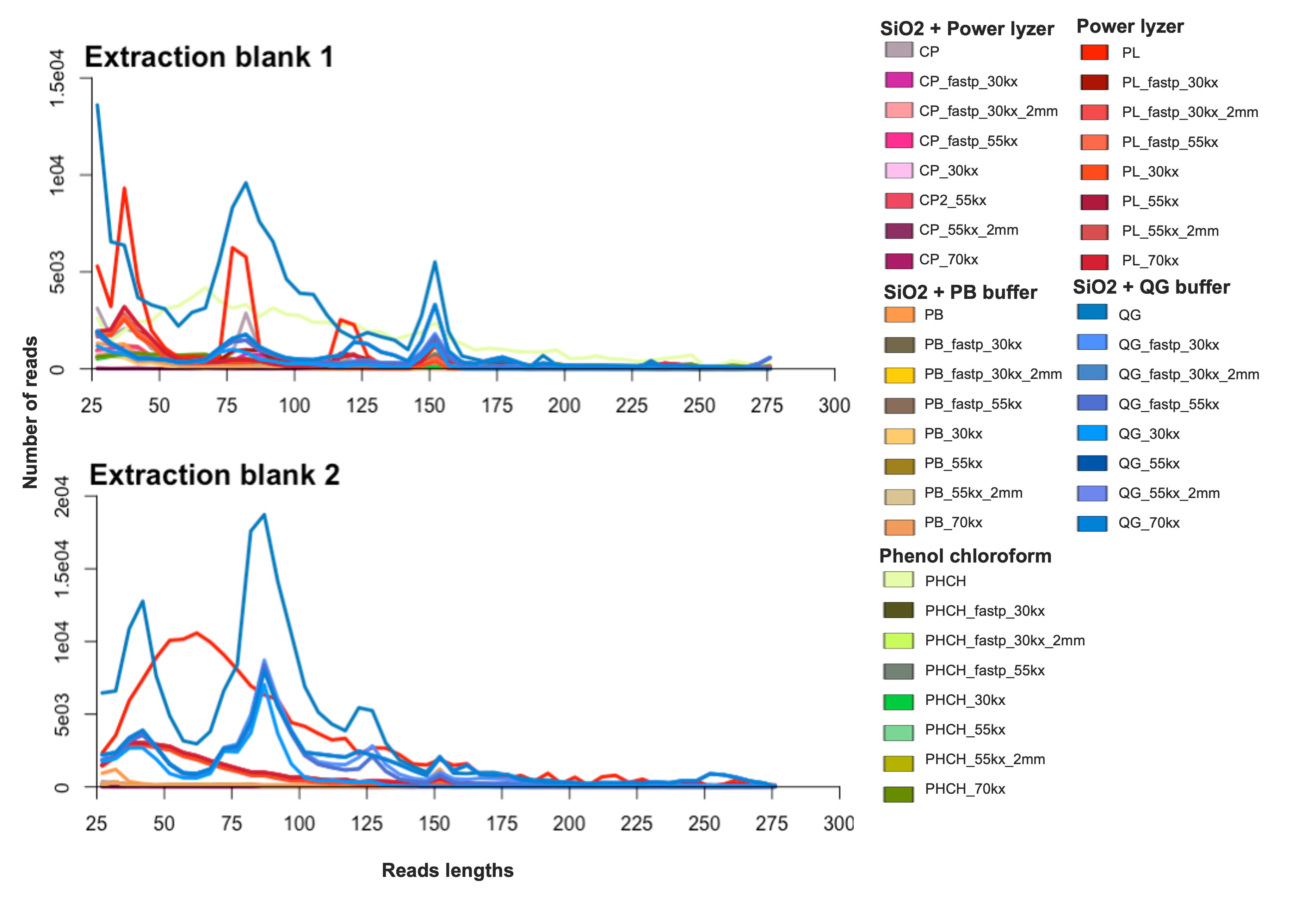

Supplement: Supplementary file 1 [file microorganisms-10-01623-s001.zip › Figure_S1.png]

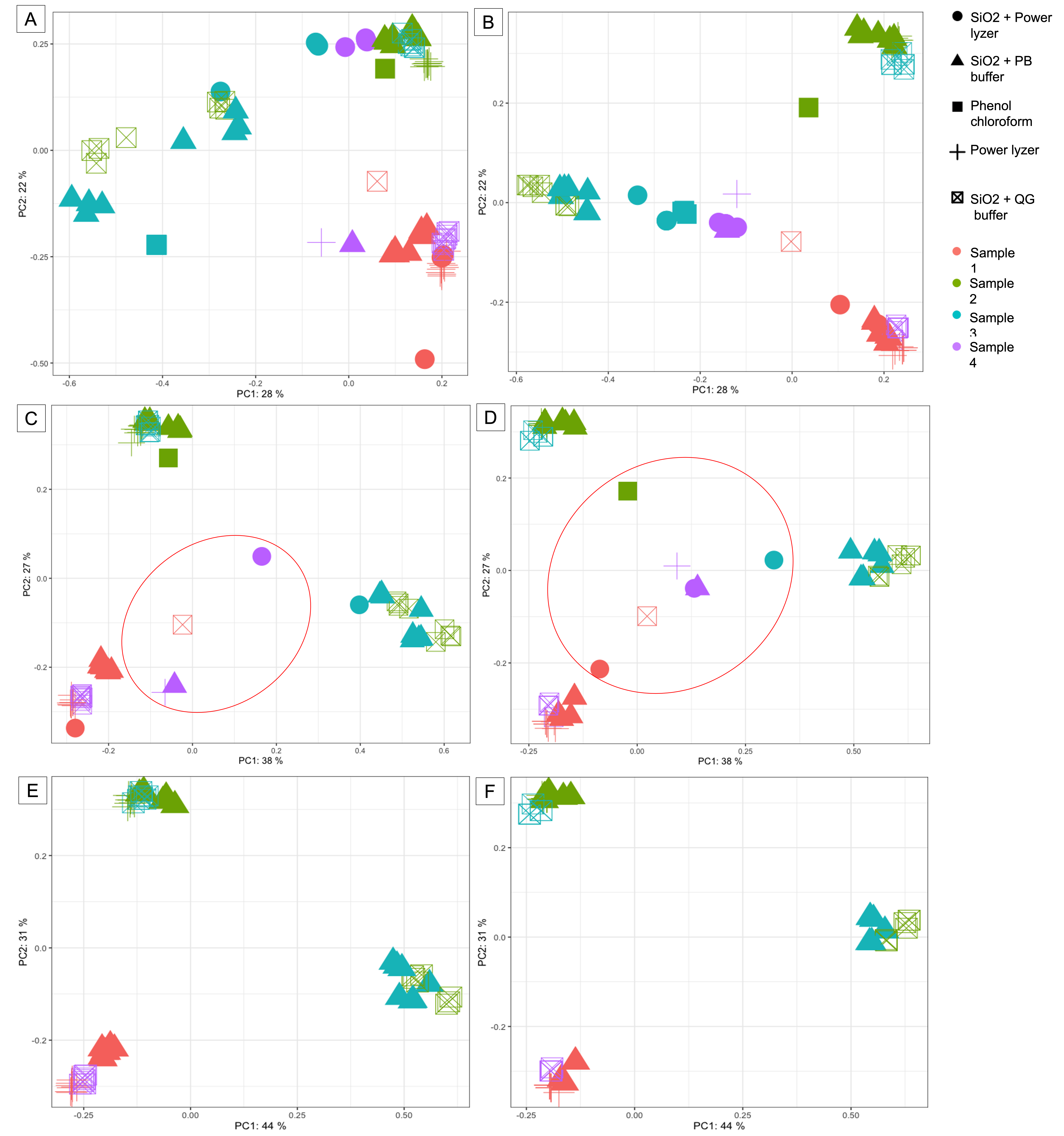

Supplement: Supplementary file 1 [file microorganisms-10-01623-s001.zip › Figure_S2.png]

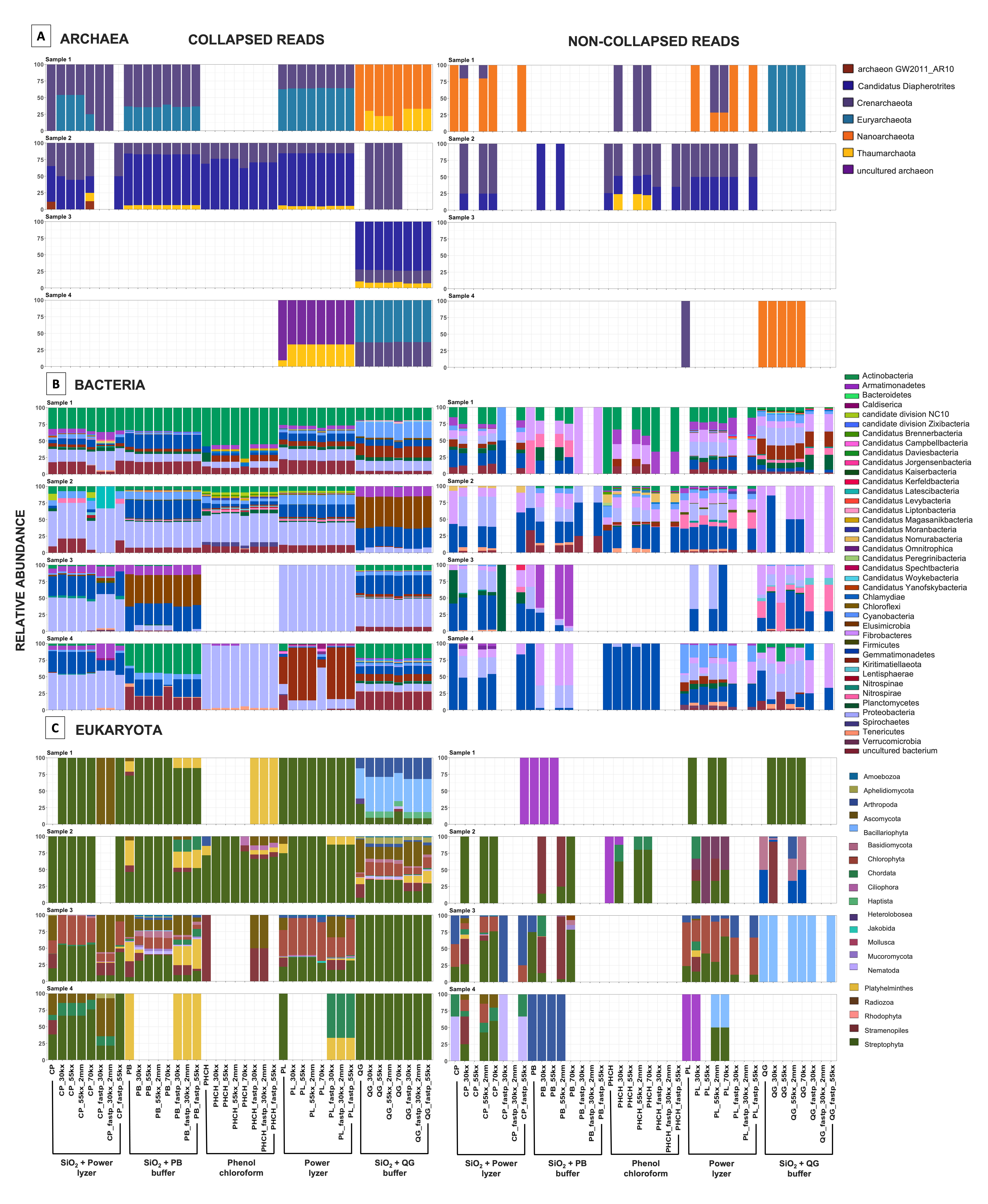

Supplement: Supplementary file 1 [file microorganisms-10-01623-s001.zip › Figure_S3.png]

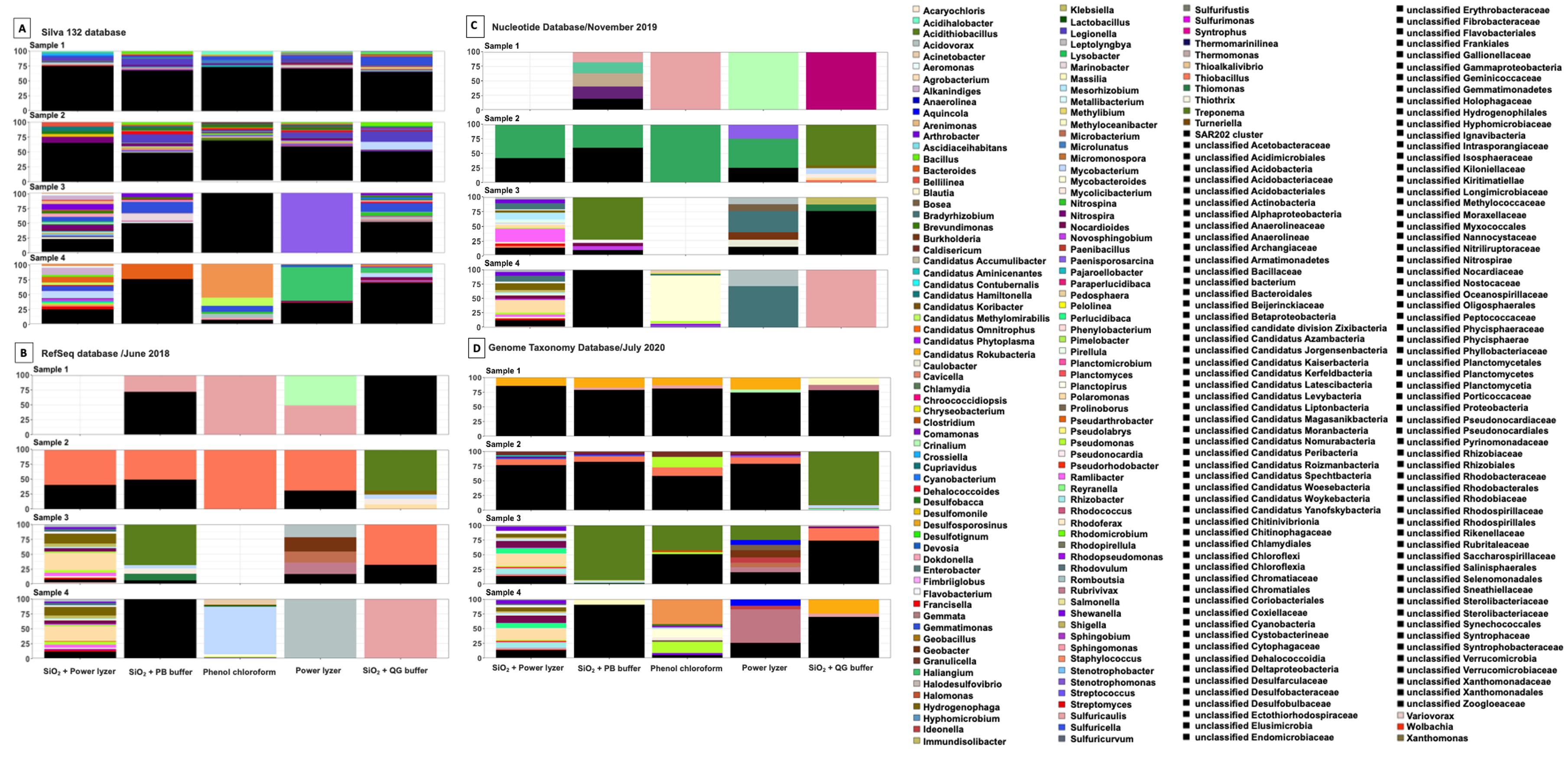

Supplement: Supplementary file 1 [file microorganisms-10-01623-s001.zip › Figure_S4.png]

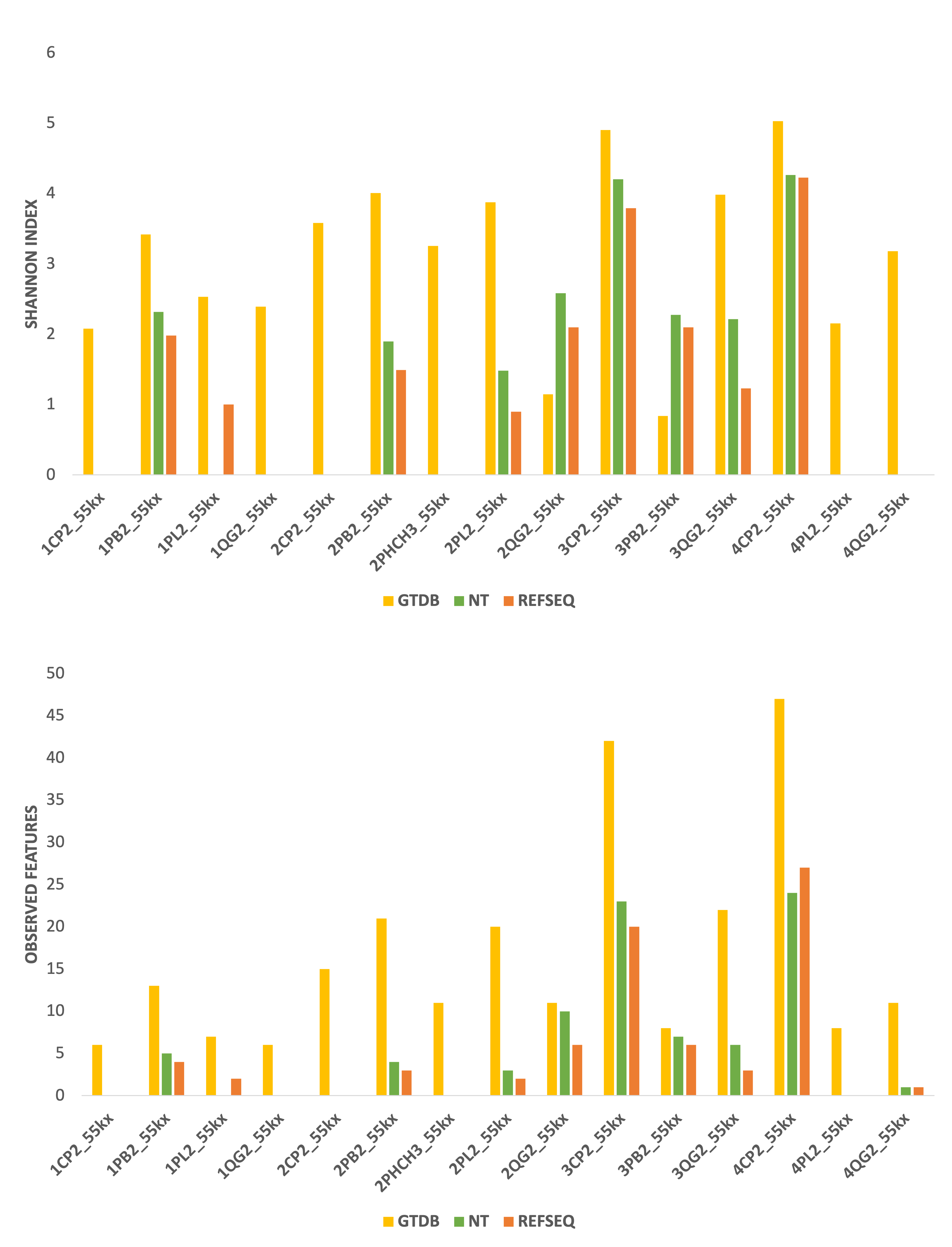

Supplement: Supplementary file 1 [file microorganisms-10-01623-s001.zip › Figure_S5.png]
